# Melatonin reverses tumor necrosis factor-alpha-induced metabolic disturbance of human nucleus pulposus cells via MTNR1B/Gαi2/YAP signaling

**DOI:** 10.7150/ijbs.65973

**Published:** 2022-03-06

**Authors:** Xianjian Qiu, Tongzhou Liang, Zizhao Wu, Yuanxin Zhu, Wenjie Gao, Bo Gao, Jincheng Qiu, Xudong Wang, Taiqiu Chen, Zhihuai Deng, Pengfei Li, Yanbo Chen, Hang Zhou, Yan Peng, Caixia Xu, Peiqiang Su, Anjing Liang, Dongsheng Huang

**Affiliations:** 1 Department of Orthopedic Surgery, Sun Yat-sen Memorial Hospital of Sun Yat-sen University, Guangzhou, Guangdong, China.; 2 Musculoskeletal Research Laboratory, Department of Orthopaedics & Traumatology, Faculty of Medicine, The Chinese University of Hong Kong, Hong Kong, China.; 3 Department of Orthopedic Surgery, The Third Affiliated Hospital of Sun Yat-sen University, Guangzhou, Guangdong, China.; 4 Department of Orthopedic Surgery, The First Affiliated Hospital of Sun Yat-sen University, Guangzhou, Guangdong, China.; 5 Research Centre for Translational Medicine, The First Affiliated Hospital of Sun Yat-sen University, Guangzhou, Guangdong, China.

**Keywords:** intervertebral disc degeneration, nucleus pulposus cells, melatonin, TNF-α, MTNR1B, yes-associated protein.

## Abstract

**Background**: Intervertebral disc degeneration (IDD), the main cause of low back pain, is closely related to the inflammatory microenvironment in the nucleus pulposus (NP). Tumor necrosis factor-α (TNF-α) plays an important role in inflammation-related metabolic disturbance of NP cells. Melatonin has been proven to regulate the metabolism of NP cells, but whether it can protect NP cells from TNF-α-induced damage is still unclear. Therefore, this study aims to investigate the role and specific mechanism of melatonin on regulating the metabolism of NP cells in the inflammatory microenvironment.

**Methods:** Western blotting, RT-qPCR and immunohistochemistry were used to detect the expression of melatonin membrane receptors (MTNR1A/B) and TNF-α in human NP tissues.* In vitro*, human primary NP cells were treated with or without vehicle, TNF-α and melatonin. And the metabolic markers were also detected by western blotting and RT-qPCR. The activity of NF-κB signaling and Hippo/YAP signaling were assessed by western blotting and immunofluorescence. Membrane receptors inhibitors, pathway inhibitors, lentiviral infection, plasmids transfection and immunoprecipitation were used to explore the specific mechanism of melatonin. *In vivo*, the rat IDD model was constructed and melatonin was injected intraperitoneally to evaluate its therapeutical effect on IDD.

**Results:** The upregulation of TNF-α and downregulation of melatonin membrane receptors (MTNR1A/B) were observed in degenerative NP tissues. Then we demonstrated that melatonin could alleviate the development of IDD in a rat model and reverse TNF-α-impaired metabolism of NP cells *in vitro*. Further investigation revealed that the protective effects of melatonin on NP cells mainly rely on MTNR1B, which subsequently activates Gαi2 protein. The activation of Gαi2 could upregulate the yes-associated protein (YAP) level, resulting in anabolic enhancement of NP cells. In addition, melatonin-mediated YAP upregulation increased the expression of IκBα and suppressed the TNF-α-induced activation of the NF-κB pathway, thereby inhibiting the catabolism of NP cells.

**Conclusions:** Our results revealed that melatonin can reverse TNF-α-impaired metabolism of NP cells via the MTNR1B/Gαi2/YAP axis and suggested that melatonin can be used as a potential therapeutic drug in the treatment of IDD.

## Introduction

Low back pain (LBP) is a common symptom that affects people of all ages and is the leading cause of disability globally. This health problem causes immense suffering, places a heavy burden on health care services, and carries enormous impact for societies and economies^1^, ^2^. Approximately 40% of LBP cases are associated with intervertebral disc degeneration (IDD), which is the major cause of degenerative disc diseases^3^, ^4^. Nucleus pulposus (NP) cells are the main cell type in normal NP tissues and are responsible for the homeostasis of the extracellular matrix (ECM)^5^. The intact ECM gives intervertebral discs special biomechanical properties and ensures the dynamic stabilization of intervertebral discs. Although the pathogenesis of IDD is still unclear currently, the impaired ECM metabolism by NP cells is thought to be the core pathological change of IDD^6^. As degeneration proceeds, the levels of inflammatory cytokines (e.g., tumor necrosis factor-α [TNF-α] and interleukin-1β [IL-1β]) produced by NP cells or immune cells in NP tissues are elevated. The inflammatory microenvironment subsequently stimulates the NP cells to reduce the synthesis of collagen type II (COL2A1) and aggrecan (ACAN), and enhance the expression of matrix metalloproteinases (MMPs) and a disintegrin-like and metalloprotease with thrombospondin type-1 motifs (ADAMTS) enzymes, leading to an imbalance between anabolic and catabolic activities of NP cells. These pathological processes induce or accelerate the development of IDD, eventually^7^.

TNF-α is one of the most studied among the IDD-related inflammatory cytokines. Besides promoting the expression of MMPs and ADAMTSs in NP cells^8^, TNF-α can also induce NP cells to produce several proinflammatory cytokines and further exacerbate the inflammatory response^9^, ^10^. In addition, TNF-α has been implicated in disc herniation and nerve irritation and ingrowth^11^, ^12^. Lai et al. reported that TNF-α intradiscal injection could increase the painful behavior in a rat IDD model^13^. Moreover, exogenous TNF-α intradiscal injection can lead to disc degeneration in a porcine model^14^. Considering the importance of TNF-α in the pathological mechanism of IDD, it is necessary to seek effective approaches to alleviate the TNF-α-induced inflammatory response and restore the balance between anabolic and catabolic activities of NP cells to prevent the development of IDD.

Melatonin is a hormone, which mainly produced by the pineal gland. Melatonin has a wide range of effects, such as the tumor inhibition, modulation of immunology and regulation of circadian rhythms^15^. In the past decades, melatonin has been proven to be a promoter of osteochondral development, metabolism and repair^16^-^18^. Several studies have shown that melatonin plays an important role in preventing osteoporosis and osteoarthritis^19^-^21^, suggesting that melatonin has extremely high potential application value in the field of degenerative diseases. In 2006, Turgut et al. [22]showed that pinealectomy in chicken could accelerate the IDD process, revealing the strong relationship between melatonin and IDD. In the past years, some studies have proven that melatonin treatment could prevent ECM degradation of NP cells via reducing oxidative stress^23^, activating autophagy^24^ or suppressing PI3K-AKT signaling^25^. However, previous studies mainly focused on the effects of melatonin on NP cells under physiological conditions, while ignoring the influence of the inflammatory response. Whether melatonin can protect the metabolism of NP cells from the inflammatory response, and in particular restore TNF-α-induced metabolic disturbance of NP cells remains unknown.

Yes-associated protein (YAP) is the final effector of the Hippo signaling pathway, which plays an important role in organ morphogenesis, cell proliferation and differentiation, and tissue homeostasis and regeneration^26^. Recently, several studies have shown that Hippo/YAP signaling may play a predominant role in the pathogenesis of orthopedic degenerative diseases, such as osteoarthritis and osteoporosis^27^. A study by Deng et al.^28^ showed that YAP activation can protect articular cartilage and YAP depletion in chondrocytes may increase cartilage destruction. Their study also demonstrated that YAP activation can attenuate the cartilage inflammatory response induced by IL-1β and TNF-α^28^. In addition, upregulation of YAP has been confirmed to have a positive effect on alleviating vascular and pulmonary inflammation^29^, ^30^, suggesting that YAP plays a vital role in the regulation of the inflammatory response in numerous cell types. However, the role of YAP in NP cells remains unclear. Whether YAP exerts protective effects on TNF-α-induced degenerative NP cells is still unknown. Although multiple studies have shown that melatonin could regulate YAP activity^31^-^33^, few studies have focused on the specific mechanism by which melatonin activates YAP. Thus, whether and how YAP plays a role in the crosstalk between melatonin and TNF-α during IDD pathogenesis deserves further research.

In our study, we explored whether melatonin could reverse TNF-α-induced metabolic disturbance of NP cells. The purposes of this research are (i) to further confirm the protective effects of melatonin as an anti-inflammatory and pro-anabolic role during IDD pathogenesis and (ii) to provide additional theoretical support for the use of melatonin in the treatment of IDD.

## Materials and methods

### Antibodies and reagents

Antibodies against collagen type II (COL2A1), aggrecan (ACAN), a disintegrin-like and metalloprotease with thrombospondin type-1 motifs 5 (ADAMTS5), matrix metalloproteinase 13 (MMP13), melatonin receptor 1A (MTNR1A), melatonin receptor 1B (MTNR1B), tumor necrosis factor-alpha (TNF-α), and GNAI2 (Gαi2) were purchased from Abcam (Cambridge, UK). Antibodies against Hippo signaling proteins (YAP, phospho-YAP [p-YAP], LATS1 and LATS2), and NF-κB signaling proteins (p65, p-p65, IκBα and p-IκBα), and goat anti-mouse IgG and goat anti-rabbit IgG secondary antibodies were purchased from Cell signaling Technology Inc (Boston, MA, USA). Antibodies against p-LATS1/2 was obtained from Affinity Biosciences LTD (OH, USA). Anti-GADPH and anti-α-tubulin was obtained from Proteintech Group Inc (Rosemont, IL, USA). Melatonin, luzindole, 4P-PODT and cycloheximide (CHX) was obtained from Sigma-Aldrich (St. Louis, MO, USA), and recombinant human TNF-α was purchased from R&D Systems (Minneapolis, MN, USA). S26131 was purchased from Med Chem Express (MCE, New Jersey, USA). Verteporfin (VP) was obtained from Selleck Chemicals (Houston, TX, USA).

### Rat model of IDD

Forty-eight female Sprague-Dawley rats (12 weeks, 250-300g) were purchased and housed in specific pathogen-free facilities on a 12/12-hours light/dark cycle. The rat IDD models were established by puncture of a caudal intervertebral disc as described by Zhang et al.^34^. Briefly, the rats were placed in the prone position after being anesthetized by 3% sodium pentobarbital (30 mg/kg) via intraperitoneal injection. The needle (21G) punctures were performed at the level of the caudal intervertebral disc 5-6 located by X-ray. The needle was inserted perpendicular to the cartilage endplate with a puncture depth of 5 mm. After rotating 360°and waiting for 1 minute, the needle was pulled out. Amikacin (10 mg/kg/day) was injected daily after surgery for 3 consecutive days to prevent infection. Magnetic resonance imaging (MRI) was performed 2 weeks after surgery, and Pfirrmann grading system was used to assess the disc degeneration^35^.

The 48 rats were divided into four groups: the control group, the puncture group, the puncture with melatonin treatment group, and the puncture with melatonin and luzindole treatment group. Melatonin (10 mg/kg) was injected intraperitoneally (i.p.) every 3 days for 4 weeks from the first day after surgery. Luzindole(1 mg/kg) was injected i.p. 1 hour before melatonin treatment to block the effects of melatonin. After reaching the endpoint of the experiment, MRI was performed to assess the degree of IDD. The rats were sacrificed, and the intervertebral disc tissues were collected for histological examination. The animal study was approved by Institutional Animal Care and Use Committee of Sun Yat-sen University (Approval No. SYSU-IACUC-2020-B0378).

### Culture and treatment of human NP cells

Human NP cells were obtained from ScienCell (Carlsbad, CA, USA). Cells were cultured at 37˚C/5% CO_2_ by using human NP cell medium (ScienCell) and the cell medium was changed every 3 days. Cell trypsinization, counting and passaging were performed when NP cells reached 80%-90% confluence. Cells from passages 3-6 were cultured in 6-well plate at a density of 1.5 × 10^6^ cells/ml. At 12h after plating, cells were given the indicated treatment for subsequent experiments.

### Human NP tissues

The degenerative NP tissues were collected from 12 patients with lumbar disc degeneration (six males and six females; mean age: 53.6±6.2 years). Control NP specimens were obtained from 6 individuals suffering from fresh traumatic lumbar fractures (three males and three females; mean age: 18.2±4.2 years). All patients underwent MRI scan before surgery, and Pfirrmann grading system was used to assess the disc degeneration. Surgical treatments of all patients were performed at Sun Yat-sen Memorial Hospital of Sun Yat-sen University (Guangzhou, China) between January 2018 and December 2019. Human NP tissues were obtained during the discectomy surgery. The clinical characteristics of the patients are listed in Supplementary Table Ⅰ.

### Histology and immunohistochemistry

Tissues were fixed with paraformaldehyde (4%) at room temperature for 24 hours before being embedded in paraffin. Paraffin sections (4-µm thick) were prepared and coated on glass slides. After deparaffinization by xylene and ethanol, hematoxylin-eosin (HE) and safranin-O staining were performed to assess the histological changes, while immunohistochemistry (IHC) was performed to assess the levels of proteins of interest. For HE staining, the tissue sections were stained with hematoxylin for 2 minutes and then with eosin for 3 minutes at room temperature. For safranin-O staining, the tissue sections were stained with safranin for 15 minutes and then with fast green dye solution for 2 minutes at room temperature. For IHC, the tissue sections were treated with pepsin at 37℃ for 15 minutes and incubated with peroxidase for 20 minutes. Tissue sections were blocking by bovine serum albumin (5%, Sigma‑Aldrich) for 30 minutes. Histostain-Plus kit from Zhongshan Golden Bridge Bio-technology (ZSGB-Bio, Beijing, China) was used to perform IHC with anti‑MTNR1A, anti- MTNR1B, anti-TNF-α, anti-YAP, and anti-p-p65 (1:200) at 4˚C overnight. Then, DAB Horseradish Peroxidase Color Development kit (ZSGB-Bio) was applied for detection of staining. Finally, all glass slides were photographed by an Olympus BX63 microscope (Olympus, Tokyo, Japan). Histological scores were assessed using the the intervertebral disc scoring system 36. Image-Pro Plus 6.0 software (Media Cybernetics, Inc., Rockville, MD, USA) was applied for semi-quantitative analysis of IHC images through integrated optical density (IOD).

### Reverse transcription‑quantitative polymerase chain reaction (RT‑qPCR)

RNA-iso Plus reagent (TaKaRa, Dalian, China) was used to extracted total RNA. Then PrimeScript RT Master Mix (TaKaRa) was used to convert RNA to cDNA. qPCR was performed to analyze the expressions of the following genes: *MTNR1A, MTNR1B, COL2A1, ACAN, MMP13, ADAMTS5, YAP, IκBα, CYR61, CTGF, MMP9, CCL2, GNAI2, TNF-α,* and* IL-1β.* Glyceraldehyde-3-phosphate dehydrogenase (GAPDH) gene expression was used as reference. *ΔCt* was obtained by subtracting the *Ct* value of GAPDH from the target gene. The average *ΔCt* value was acquired by three independent experiments. The 2^-ΔΔCt^ method was used to determine the relative expression levels of each gene. Primer sequences are listed in Supplementary Table II.

### Western blot analysis

Adherent cells were lysed in RIPA lysis buffer (Beyotime, Shanghai, China) containing protease inhibitor cocktail (Biotool, Houston, TX, USA). NP tissues were ground after adding RIPA lysis buffer. Sample proteins were obtained by centrifugation of cell or tissues lysates. BCA protein assay kit (Beyotime) was used to quantify the protein concentration of each sample. SDS-polyacrylamide gel electrophoresis (PAGE) were performed to separate specific proteins. Then, the specific proteins were transferred to PVDF membranes (Millipore). 5% nonfat dry milk was applied as blocking reagent of membranes for 60 minutes. Then, incubating membranes with the designated antibodies were performed at 4°C overnight. Then, secondary antibodies were used to incubate the membranes and protein bands were visualized with an ECL kit (Millipore).

### Degradation assay

Human NP cells were seeded in 12-well plates and pretreated with indicated treatment for 24 hours. After adding cycloheximide (CHX, 5 μM), protein lysates were prepared at indicated time points. SDS-PAGE was performed to separate proteins. Levels of YAP at indicated time points were determined by western blot analysis. ImageJ software (National Institutes of Health, Bethesda, MD, USA) was used to quantify the bands and GAPDH was used as the control to normalize the bands.

### Immunoprecipitation

The immunoprecipitation (IP) was performed as described by Lian et al.^19^. Briefly, cells were treated with indicated treatment for 2 hours. Then, cells were lysed with immunoprecipitation lysis buffer. After quantification, proteins from each sample were incubated with anti-MTNR1B and protein A/G beads (MCE) overnight at 4°C. The beads were washing with immunoprecipitation wash buffer seven times and the immunoprecipitates on the beads were collected by centrifugation and analyzed by western blot.

### Cell transduction

*YAP*-shRNA, *YAP*-FLAG, ubiquitin-HA and *Gαi2*-shRNA plasmids were constructed by Genechem (Shanghai, China). Cells were transfected by using Lipofectamine 3000 (Invitrogen, Carlsbad, CA, USA). To knock down MTNR1A and MTNR1B, the pLKD-CMV-mcherry-2A-Neo-U6-shRNA (MTNR1A) and pLenti-U6-spgRNAv2.0-CMV-sfGFP-spCas9 (MTNR1B) were constructed by OBIO (Shanghai, China). Lentiviral infection was conducted as described previously^19^. Briefly, human NP cells were seeded in 6-well plate overnight at a density of 10^5^ cells per well with complete medium. Then viral supernatant and polybrene (5 μg/ml) were used to treat the cells for transfection. After 24 hours of treatment, normal medium was changed for cell culture for 72 hours. The transfection efficiencies were detected by fluorescence observed by fluorescence microscope (Leica 4000MB, Japan). The transfected cells were harvested after adding the specific treatment for western blot and RT-qPCR analyses.

### Immunofluorescence

Human NP cells were seeded on cover glasses in 12-well plates and underwent different treatment. After 24 hours of treatment, paraformaldehyde (4%) was applied for cell fixation for 20 minutes at room temperature. The 0.5% Triton X-100 was used to permeabilize the cells for 20 minutes. Goat serum was applied to block the cells for 30 minutes. Then, designated antibodies incubation was performed at 4℃ overnight. After incubating with the fluorescein-conjugated secondary antibodies, the cells were photographed with an Olympus BX63 microscope (Olympus, Tokyo, Japan).

### Statistical analysis

All quantitative data were presented as the mean ± SD. Two-tailed paired Student's *t* tests were performed to access the comparisons between groups. SPSS 20.0 statistical software package (SPSS, Inc., Chicago, IL, USA) was applied to fulfill the statistical analyses. *P* < 0.05 means statistically significant.

## Results

### Expression of melatonin receptors is downregulated in degenerative human NP tissues

**Figure 1:**
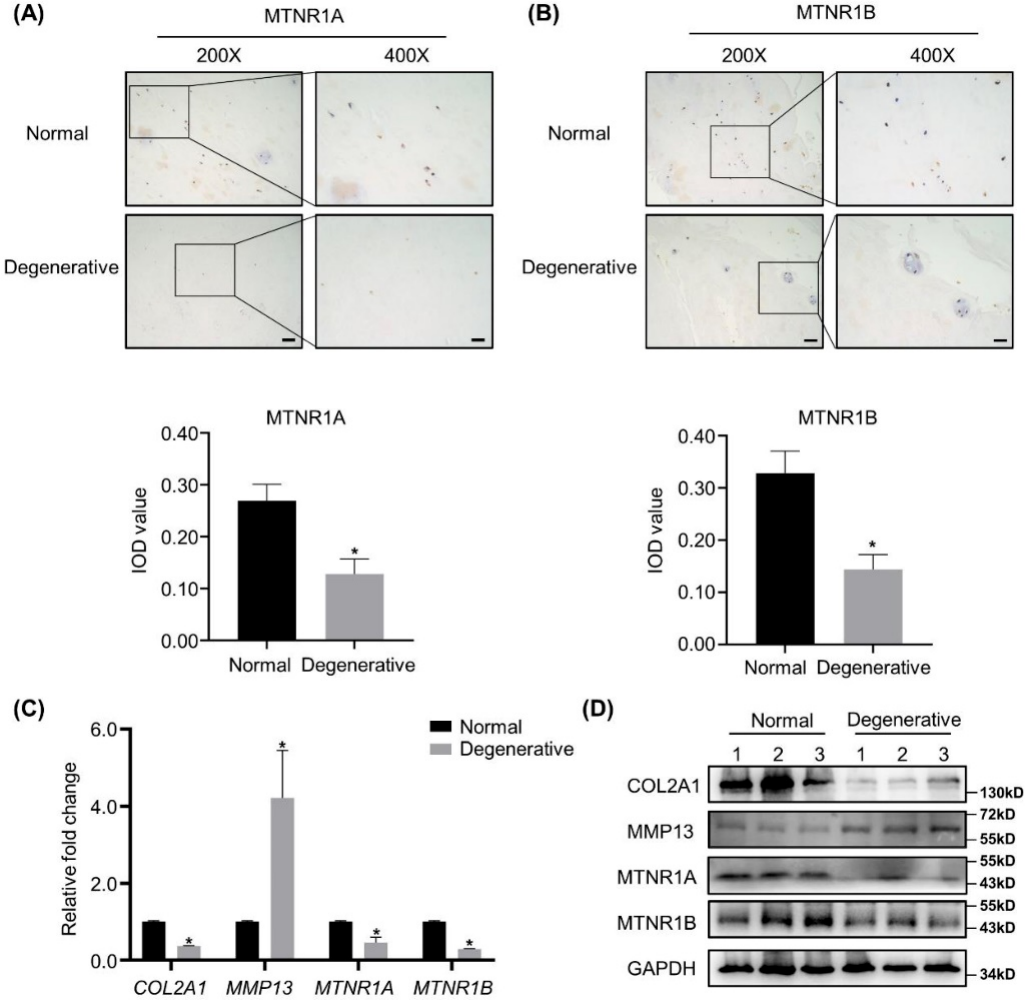
** Melatonin receptors are downregulated in degenerative human NP tissues. (A and B)** The expression of melatonin receptors MTNR1A and MTNR1B in human normal and degenerative NP tissues were assessed by IHC. Representative images of different magnifications were displayed. Scale bars: 100 μm and 50 μm for 200× and 400× images, respectively. Integral optical density (IOD) values were measured from IHC images as the semi-quantitative analysis of MTNR1A and MTNRR1B protein expression. **(C and D)** The expression of COL2A1, MMP13, MTNR1A and MTNR1B in human normal and degenerative NP tissues (*n*=3) were measured by RT-qPCR **(C)** and WB **(D)**. The results in **(C)** and **(D)** were representative of three independent experiments. *P < 0.05.

IHC analyses of MTNR1A and MTNR1B, the membrane receptors of melatonin, were conducted in 12 degenerative human NP tissues and 6 corresponding normal specimens. As shown in Figure 1A and B, the expression of MTNR1A and MTNR1B was downregulated in the degenerative NP tissues. Semi-quantitative analysis of MTNR1A and MTNR1B was shown below the respective images. RT-qPCR, WB analyses and quantification revealed that compared with the nondegenerative control, melatonin receptor levels were significantly decreased in degenerative NP tissues at both mRNA and protein level (Figure 1C, D and Supplementary Figure 1C, D). We also detected the expression of anabolic and catabolic markers (COL2A1 and MMP13) and found that compared with the controls, the expression of COL2A1 was downregulated, while the expression of MMP13 was upregulated in degenerative NP tissues (Figure 1C, D and Supplementary 1A, B).

### Melatonin can alleviate the development of IDD in the rat model

**Figure 2:**
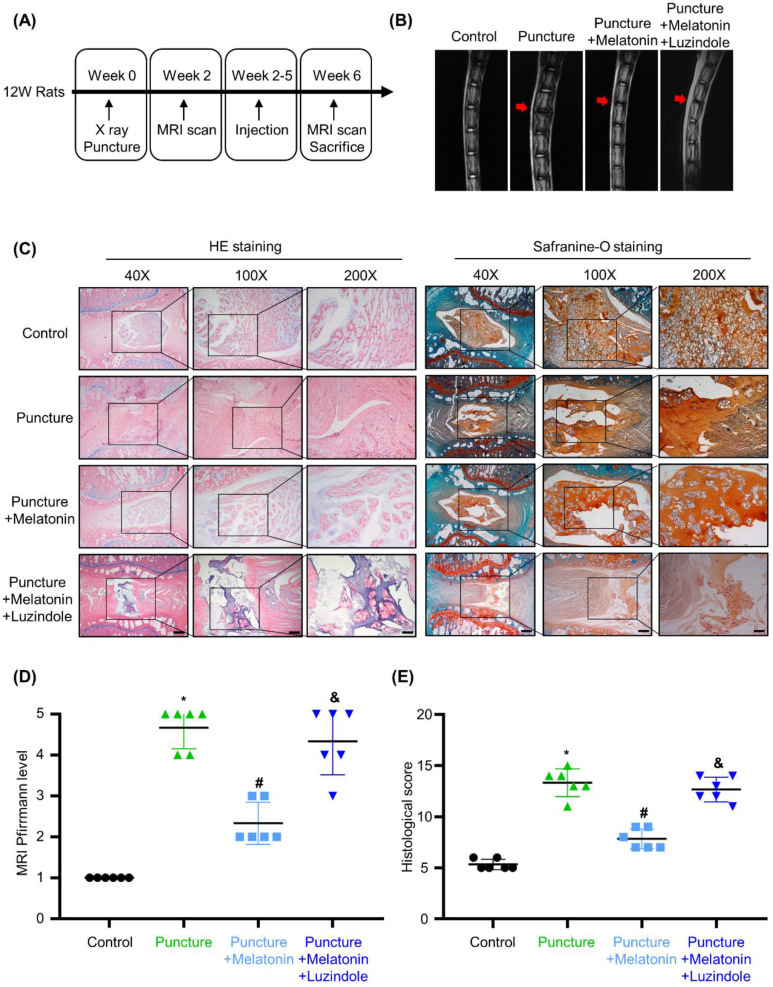
**Melatonin can alleviate the development of IDD in a rat model. (A)** A Schematic of the *in vivo* experiment. Two weeks after puncture of the caudal intervertebral disc at Co5/6, rats were i.p. injected with melatonin or both melatonin and luzindole every 3 days for 4 weeks (*n*=12 for per group). **(B)** T2-weighted MRI scans of the caudal intervertebral disc of rats after no puncture, puncture, puncture with melatonin (10 mg/kg), or puncture with both melatonin and luzindole (1 mg/kg). Red arrows indicate the affected intervertebral discs. **(C)** HE staining and safranin O staining of the affected intervertebral disc of rats treated with indicated treatments. Representative images of different magnifications were displayed. Scale bars: 500 μm, 200 μm and 100 μm for 40×, 100× and 200× images, respectively. **(D)** Pfirrmann score analysis of T2-weighted MRI images of the caudal intervertebral disc of rats followed by indicated treatments (*n*=6 for per group). **(E)** Histological scores of the affected intervertebral discs of rats treated with indicated treatments (*n*=6 for per group). * means *P* < 0.05 compared with the control group. # means* P* < 0.05 compared with the puncture group. & means* P* < 0.05 compared with the puncture with melatonin group.

To investigate the effects of melatonin *in vivo*, we constructed an IDD rat model by puncture of caudal intervertebral disc. A schematic of the *in vivo* experiment is shown in Figure 2A. Two weeks after surgery, melatonin was i.p. injected every 3 days. And luzindole, a melatonin receptors antagonist (MTNR1A/B-nonselective), was i.p. injected 1 hours before melatonin treatment as a pre-treatment to block the effects of melatonin. After 4 weeks of treatment, as shown in Figure 2B and D, the T2-weighted MRI images and Pfirrmann scores revealed that after the puncture, degeneration was observed in the caudal intervertebral disc. With melatonin treatment, the degeneration of the affected intervertebral disc was significantly relieved. Further, luzindole pre-treatment could block the protective effect of melatonin, suggesting that melatonin relies on its membrane receptors to exert its protective effects in IDD. HE staining, safranine-O staining and the histological score reveal that in the puncture group, the structure of the intervertebral disc was disordered, accompanied by a serious loss of matrix in the NP. And an unclear boundary between NP and annulus fibrosus was observed in the degenerative discs (Figure 2B). Melatonin treatment protected the intervertebral disc from damage and could restore matrix synthesis in the NP tissue to a certain extent, but this protective effect could be blocked by luzindole pre-treatment (Figure 2B and E). These results revealed that melatonin could alleviate the development of IDD *in vivo* and that this effect was mostly mediated by its membrane receptors.

### Melatonin can restore the balance between anabolism and catabolism of NP cells in the presence of TNF-α *in vitro*

**Figure 3:**
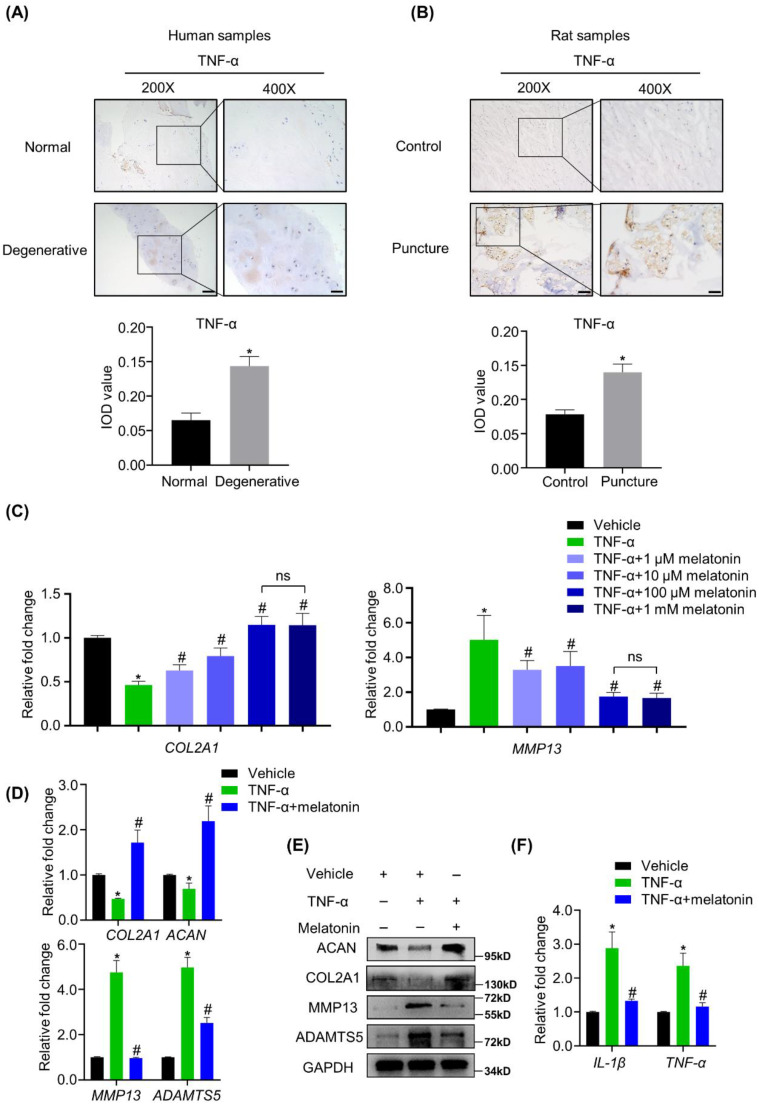
** Melatonin restores the TNF-α-induced metabolic disturbance of NP cells *in vitro*. (A and B)** The expression of TNF-α in NP tissues from human and rat were detected by IHC. Representative images of different magnifications were displayed. Scale bars: 100 μm and 50 μm for 200× and 400× images, respectively. **(C)** RT-qPCR analysis was conducted to assess the mRNA expression of COL2A1 and MMP13 in human NP cells with the addition of vehicle(ethanol), TNF-α (10 ng/ml) and a gradient concentration of melatonin for 48 hours to determine the optimum concentration. After indicated treatment for 48 hours, RT-qPCR **(D)** and WB **(E)** analyses were conducted to assess the expression of COL2A1, ACAN, MMP13, and ADATMS5 in human NP cells. RT-qPCR **(F)** analysis was conducted to assess the expression of IL-1β and TNF-α. * means *P* < 0.05 compared with the vehicle group. # means *P* < 0.05 compared with the TNF-α group. Ns means not significant.

We found that TNF-α was highly expressed in both human and rat degenerative NP tissues (Figure 3A and B), strongly suggesting that TNF-α is associated with the matrix metabolism disorders of NP cells. To investigate whether melatonin can protect NP cells from TNF-α-induced metabolic disturbance, human NP cells were treated with or without vehicle, 10 ng/ml TNF-α and melatonin. Our preliminary experiments show that compared with the other concentrations (1 μM, 10 μM and 1 mM) of melatonin, the concentration of 100 μM showed the best protective effects against TNF-α (Figure 3C). RT-qPCR and WB analyses revealed that TNF-α treatment downregulated the expression levels of COL2A1 and ACAN and upregulated the expression levels of MMP13 and ADAMTS5. After adding 100 μM melatonin, the destructive effect of TNF-α was reversed (Figure 3D and E). Melatonin treatment also downregulated the TNF-α-induced expression of inflammatory mediators, such as IL-1β and TNF-α (Figure 3F). These results revealed that melatonin can not only protect the metabolism of NP cells, but also attenuate the inflammatory response induced by TNF-α.

### Melatonin reverses TNF-α-impaired metabolic activities of NP cells via MTNR1B

**Figure 4:**
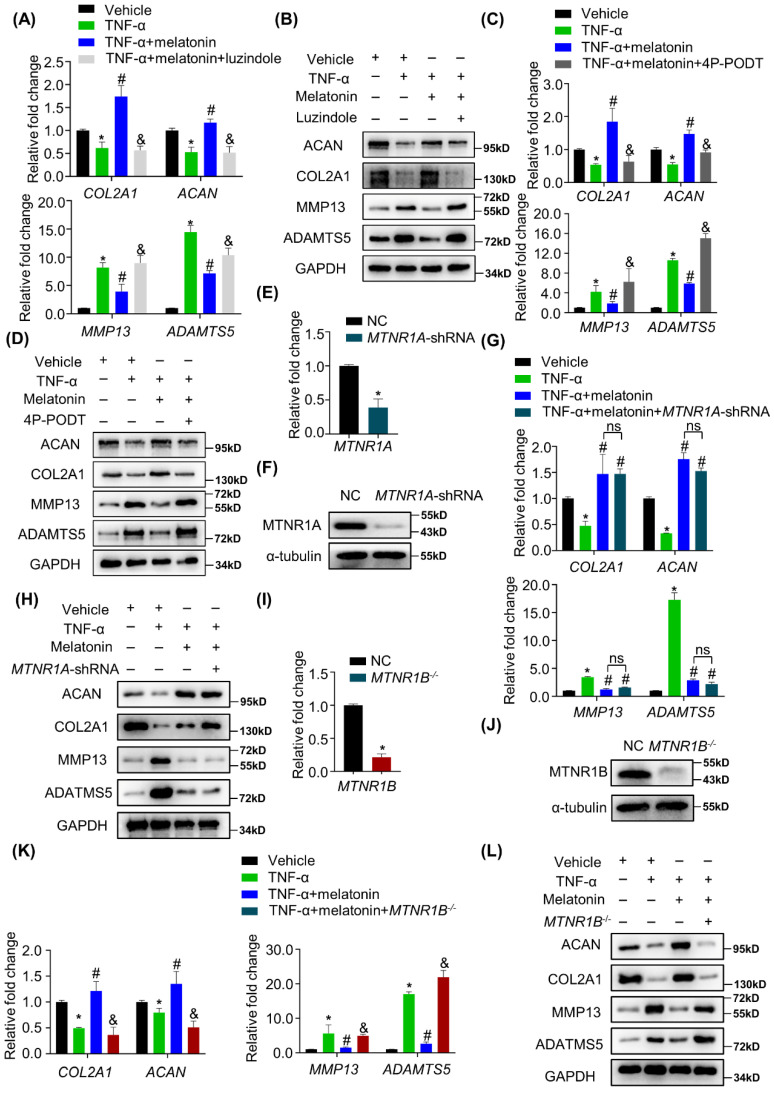
** Melatonin reverses TNF-α-impaired metabolic activities of NP cells via MTNR1B**. RT-qPCR **(A)** and WB **(B)** analyses were performed to assess the expression of COL2A1, ACAN, MMP13, and ADAMTS5 in human NP cells with or without pretreatment of luzindole (5 μM) for 1 hour followed by treatment with vehicle (ethanol), TNF-α (10 ng/ml), and melatonin (100 μM) for 48 hours. Then, RT-qPCR **(C)** and WB** (D)** analyses were performed to assess the expression of COL2A1, ACAN, MMP13, and ADAMTS5 in human NP cells with or without pretreatment with 4P-PODT (10 μM) for 1 hour before the treatment of vehicle (ethanol), TNF-α (10 ng/ml), or melatonin (100 μM) for 48 hours. *MTNR1A* was silenced by lentivirus-mediated transfection of *MTNR1A*-shRNA in human NP cells. RT-qPCR **(E)** and WB **(F)** analyses were performed to confirm the silencing efficiency. Then, NP cells were treated with indicated treatment and the expression of anabolic and catabolic markers were detected by RT-qPCR**(G)** and WB**(H)**. *MTNR1B* was deleted by lentivirus-mediated transfection of *MTNR1B*-Cas9 in NP cells, and RT-qPCR **(I)** and WB **(J)** analyses were conducted to confirm the depletion efficiency. Then, NP cells were treated with indicated treatment and the expression of anabolic and catabolic markers were detected by RT-qPCR **(K)** and WB **(L)**. * means *P* < 0.05 compared with the vehicle group. # means *P* < 0.05 compared with the TNF-α group. & means *P* < 0.05 compared with the TNF-α and melatonin group. Ns means not significant.

The most common way for melatonin to exert its biological functions is to activate its membrane receptors, including MTNR1A and MTNR1B. Whether the melatonin membrane receptors involved in the protective effects of melatonin on the metabolism of NP cells remains unknown. Therefore, cells were pretreated with luzindole before the addition of melatonin. As shown in Figure 4 A and B, TNF-α-induced metabolic disturbance was reversed by melatonin, but luzindole significantly blocked the effects of melatonin. These results revealed that melatonin membrane receptors are indeed involved in this biological process. To further clarify which receptor is involved, we used MTNR1A selective antagonist S26131 and MTNR1B selective antagonist 4P-PODT to clarify the role of MTNR1A/B. We found that after treatment with 4P-PODT, melatonin could not rescue the metabolic imbalance of NP cells in the presence of TNF-α (Figure 4C and D), which is consistent with the results after luzindole treatment. However, S26131 could not block the effects of melatonin (Supplementary Figure 2A and B). These results suggest that melatonin may rely on MTNR1B to function. We subsequently constructed *MTNR1A*-shRNA lentivirus to knock down *MTNR1A* (Figure 4E and F) and *MTNR1B*-cas9 lentivirus to knock out *MTNR1B* in NP cells (Figure 4I and J). We found that MTNR1A knockdown could not block the effects of melatonin (Figure 4G and H). By contrast, MTNR1B deletion could significantly impair the protective effects of melatonin (Figure 4K and L), which indicated that MTNR1B, but not MTNR1A, mediates the effects of melatonin on restoring TNF-α-impaired metabolic activities of NP cells.

### YAP mediates melatonin-induced protective effects on NP cells in the presence of TNF-α

**Figure 5:**
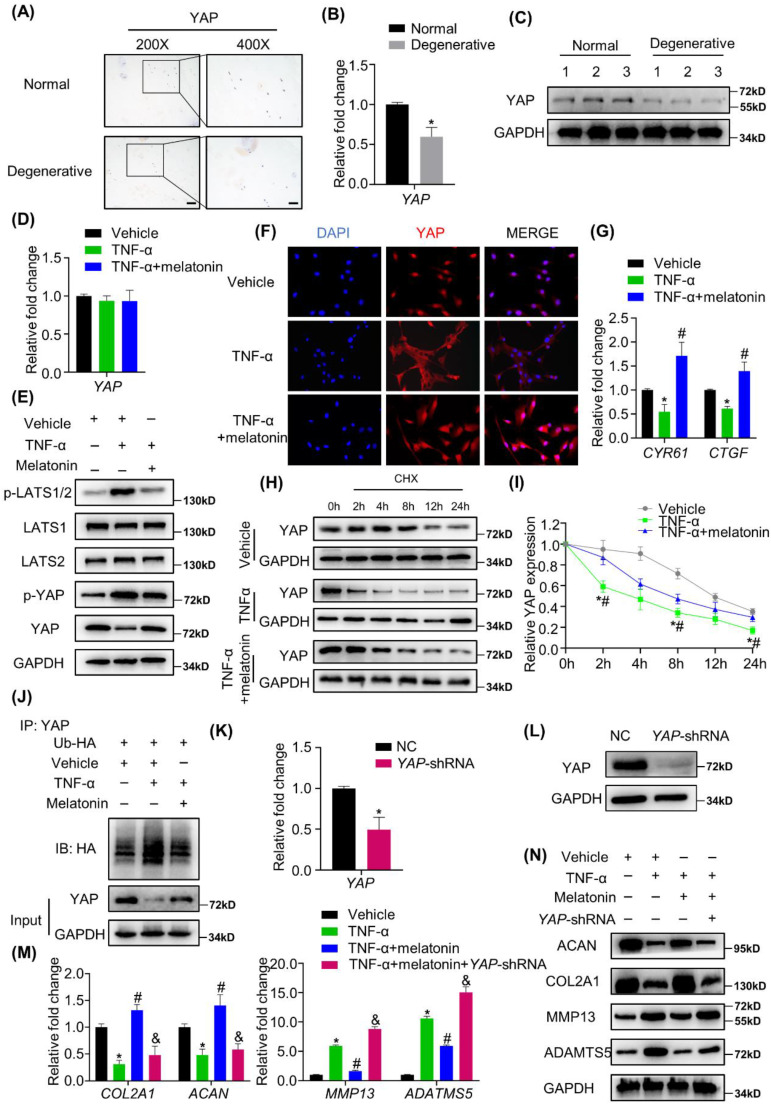
** YAP mediates melatonin-induced protective effects on NP cells in the presence of TNF-α. (A)** The expression of YAP in human NP tissues was detected by IHC. Representative images of different magnifications were displayed. Scale bars: 100 μm and 50 μm for 200× and 400× images, respectively. **(B)** RT-qPCR quantification of mRNA expression of YAP in human normal and degenerative NP tissues. **(C)** WB analysis of the protein expression of YAP in human normal and degenerative NP tissues. **(D)** RT-qPCR quantification of mRNA expression of YAP in human NP cells following the indicated treatment. **(E)** WB assay of Hippo/YAP signaling proteins in NP cells following the indicated treatment. **(F)** Immunofluorescence for the nuclear translocation of YAP protein in NP cells following the indicated treatment. Scal bar: 50 μm. **(G)** RT-qPCR quantification of mRNA expression of YAP-target genes in NP cells. **(H)** WB assay of YAP protein levels in NP cells after pretreatment with CHX (5 μM) for 2 hours followed by indicated treatment for the different time. **(I)** Quantitative analysis of degradation assay in **(H)**. **(J)** After transfection of ubiquitin-HA (Ub-HA) plasmid, ubiquitination analysis of YAP was conducted in NP cells treat with indicated treatment for 4 hours. YAP were silenced by transfection of the *YAP*-shRNA plasmid. RT-qPCR **(K)** and WB **(L)** analyses were conducted to confirm the silencing efficiency. Then, NP cells were treated with indicated treatment. The expression of anabolic and catabolic markers were detected by RT-qPCR **(M)** and WB**(N)**. * means *P* < 0.05 compared with the vehicle group. # means *P* < 0.05 compared with the TNF-α group. & means *P* < 0.05 compared with the TNF-α and melatonin group.

Whether the Hippo/YAP pathway is involved in IDD still remains unknown. We found that in human degenerative NP tissues, YAP expression was significantly decreased at both mRNA and protein levels (Figure 5 A-C and Supplementary Figure 3A). We further investigated the activity of Hippo/YAP to determine whether this pathway mediates the actions of melatonin against TNF-α *in vitro*. As shown in Figure 5D, there was no significant change in the mRNA expression levels of YAP with or without TNF-α and melatonin treatment. However, at the protein level, TNF-α treatment can significantly promote the cascade phosphorylation of the Hippo pathway, which ultimately leads to an increase in the phosphorylation level of YAP. Melatonin treatment can significantly reduce this trend (Figure 5E). We also found that melatonin can upregulate the YAP protein level in the IDD rat model (Supplementary Figure 3B). Moreover, immunofluorescence showed that TNF-α can inhibit the nuclear translocation of YAP, while melatonin can significantly reverse its translocation (Figure 5F). RT-qPCR analysis revealed that TNF-α decreased the mRNA expression of typical YAP-targeted genes, such as *CYR61* and *CTGF,* and melatonin can reverse the TNF-α-induced downregulation of YAP- targeted genes (Figure 5G).

Both TNF-α and melatonin could affect the protein level, but not the mRNA level of YAP, which indicated that they might affect the protein degradation process of YAP. We then examined the degradation kinetics of YAP in NP cells treated with or without vehicle, TNF-α and melatonin. The results revealed that TNF-α can promote the degradation of YAP, and melatonin treatment can reduce the rate of YAP degradation (Figure 5H and I). Furthermore, the ubiquitination assay showed that TNF-α can significantly increase the ubiquitination of YAP, while melatonin protects YAP from ubiquitination and degradation (Figure 5J).

To further investigate the functions of YAP mediating the effects of TNF-α and melatonin on metabolic activities in NP cells, VP, which can bind to YAP and inhibit its interaction with TEADs, was used to block the effects of YAP. After VP treatment, the reversal effect of melatonin on TNF-α was immediately blocked (Supplementary Figure 3C and D). Then, *YAP* was silenced by transfection of the *YAP*-shRNA plasmid in NP cells (Figure 5K and L), and *YAP* silencing was demonstrated to inhibit the effects of melatonin on restoring the metabolism of NP cells (Figure 5M and N), which indicated that melatonin antagonized TNF-α-induced metabolism disturbance of NP cells though maintaining YAP protein stability.

### Melatonin-mediated YAP upregulation attenuates TNF-α-induced NF-κB pathway activation by enhancing the expression of IκBα protein

**Figure 6:**
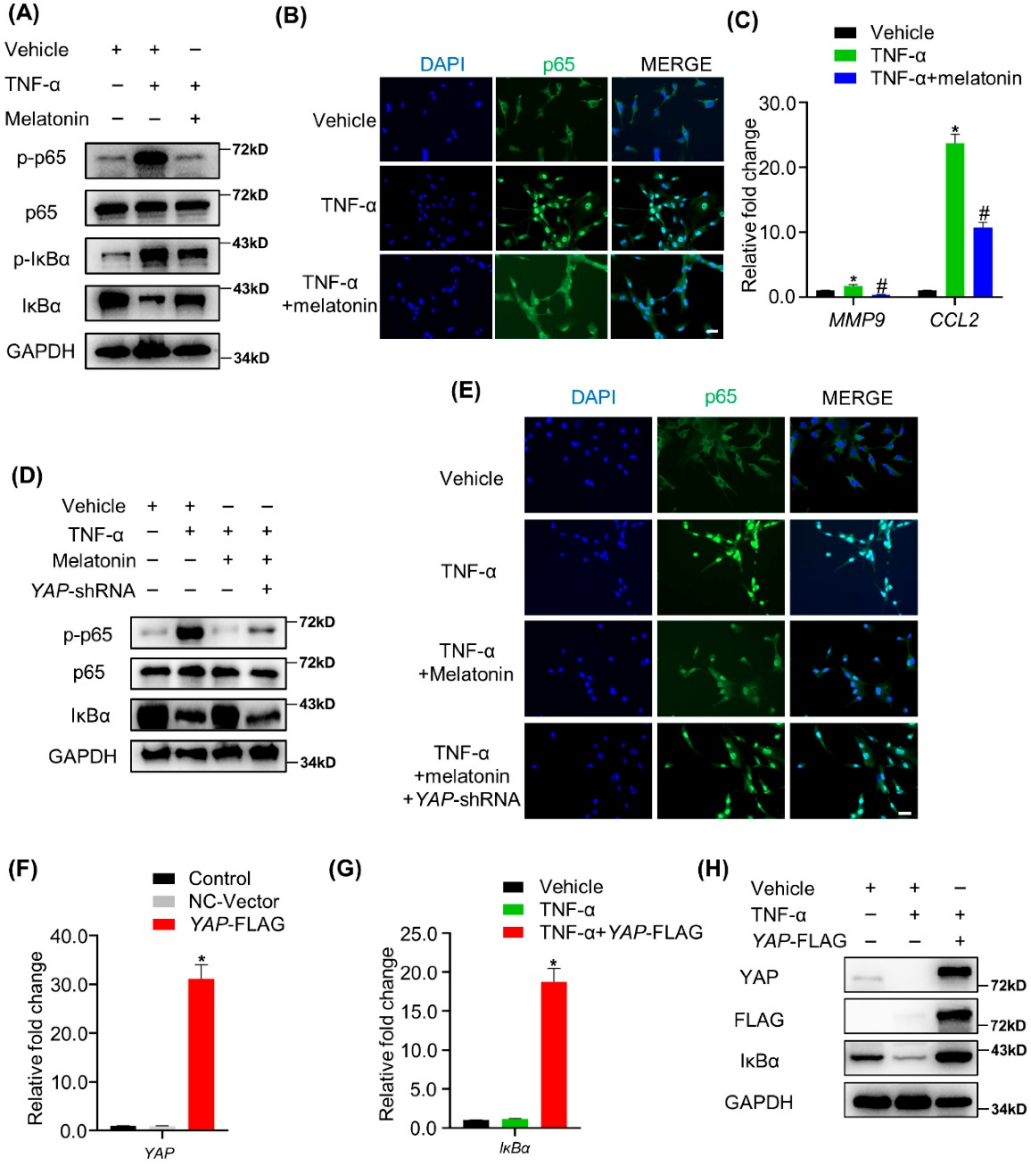
** Melatonin-mediated YAP upregulation attenuates TNF-α-induced NF-κB pathway activation by enhancing the expression of IκBα protein.** (**A**)WB assay of NF-κB pathway proteins in NP cells after treatment with vehicle (ethanol), TNF-α (10 ng/ml), and melatonin (100 μM) for 4 hours. (**B**) Immunofluorescence for the nuclear translocation of p65 protein in NP cells following the indicated treatment for 4 hours. (**C**) RT-qPCR quantification of mRNA expression of NF-κB target genes in NP cells. (**D**) WB assay of NF-κB pathway proteins in NP cells after YAP silencing followed by indicated treatment for 4 hours. (**E**) Immunofluorescence analysis of the nuclear translocation of p65 protein in NP cells after YAP silencing followed by the indicated treatment for 4 hours. YAP overexpression was conducted by transfection of the *YAP*-FLAG plasmid in NP cells. RT-qPCR (**F**) and WB (**H**) were conducted to confirm the overexpression efficiency. RT-qPCR (**G**)and WB (**H**) assay of IκBα expression levels in YAP overexpressing NP cells with or without TNF-α (10 ng/ml) treatment for 4 hours. * means *P* < 0.05 compared with the vehicle group. # means *P* < 0.05 compared with the TNF-α group. & means *P* < 0.05 compared with the TNF-α and melatonin group.

The NF-κB signaling pathway is the main downstream pathway of TNF-α, and its activation is an important cause of matrix degradation in NP^37^. In NP cells, treatment with TNF-α significantly upregulated the phosphorylation of NF-κB pathway-related proteins, including p65 and IκBα, while the addition of melatonin attenuates the activation of the NF-κB pathway (Figure 6A). Meanwhile, melatonin also suppressed the nuclear translocation of p65 protein and decreased the expression of NF-κB pathway-targeted genes in the presence of TNF-α (Figure 6B and C). IHC showed that melatonin also downregulated the expression levels of p-p65 in the rat IDD model (Supplementary Figure 4). Notably, the total expression of IκBα was downregulated upon treatment with TNF-α, while it was upregulated upon treatment with melatonin in NP cells (Figure 5A), suggesting that IκBα might be the key factor through which melatonin inhibits TNF-α-induced NF-κB pathway activation in NP cells.

YAP has been proven to have anti-inflammatory effects in a variety of cell types. Therefore, we speculated that YAP might mediate the inhibitory effects of melatonin on the NF-κB pathway. Our study showed that YAP silencing attenuated the inhibition of the NF-κB pathway by melatonin (Figure 6D). In addition, p65 was retranslocated into the nucleus upon treatment with TNF-α and melatonin when YAP was silenced (Figure 6E). On the contrary, overexpression of YAP significantly inhibited TNF-α-induced IκBα degradation (Figure 6G-I). These data strongly suggest that YAP plays an important role in melatonin's inhibition of NF-κB pathway activation caused by TNF-α and is closely related to the upregulation of the pathway inhibitory protein IκBα.

### MTNR1B-binding protein Gαi2 mediates YAP activation and the protective effects of melatonin

**Figure 7:**
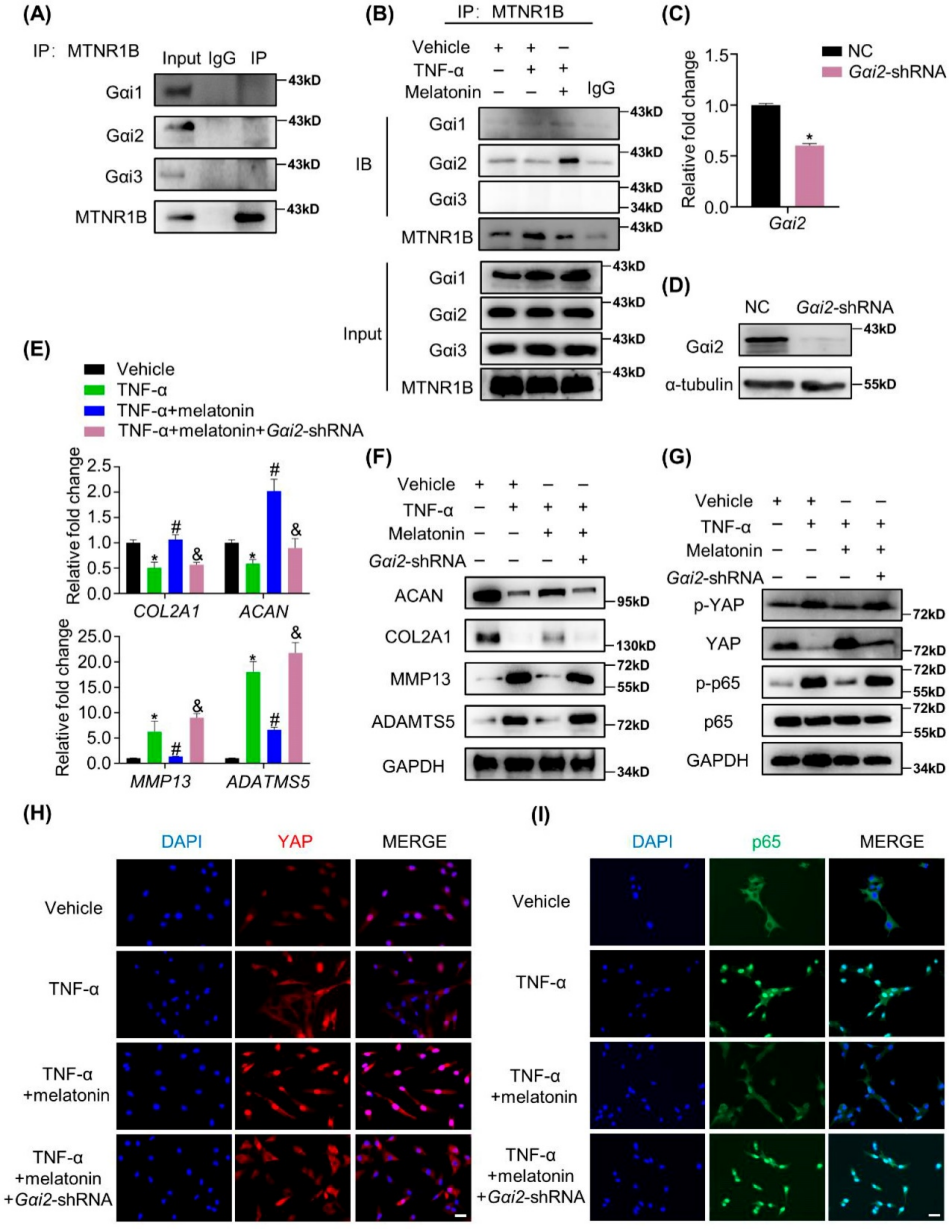
** The MTNR1B-binding protein Gαi2 mediates YAP activation and the protective effects of melatonin. (A)** Immunoprecipitation was performed to assess the endogenous interaction of MTNR1B with Gαi1, Gαi2, and Gαi3 in NP cells. **(B)** Immunoprecipitation was performed to assess the interaction of MTNR1B with Gαi1, Gαi2, and Gαi3 in NP cells treat with indicated treatment for 2 hours. Gαi2 were silenced by transfection of the *Gαi2*-shRNA plasmid. RT-qPCR **(C)** and WB **(D)** analyses were conducted to confirm the silencing efficiency. Then, NP cells were treated with indicated treatment and the expression of anabolic and catabolic markers were detected by RT-qPCR **(E)** and WB **(F)**. **(G)** WB analysis of the protein levels of YAP and p65 after Gαi2 silencing for 24 hours followed by indicated treatment for 4 hours. **(H)** Immunofluorescence analysis of the nuclear translocation of YAP in NP cells after Gαi2 silencing followed by the indicated treatment for 4 hours. **(I)** Immunofluorescence analysis of the nuclear translocation of p65 in NP cells after Gαi2 silencing followed by the indicated treatment for 4 hours. * means *P* < 0.05 compared with the vehicle group. # means *P* < 0.05 compared with the TNF-α group. & means *P* < 0.05 compared with the TNF-α and melatonin group.

**Figure 8:**
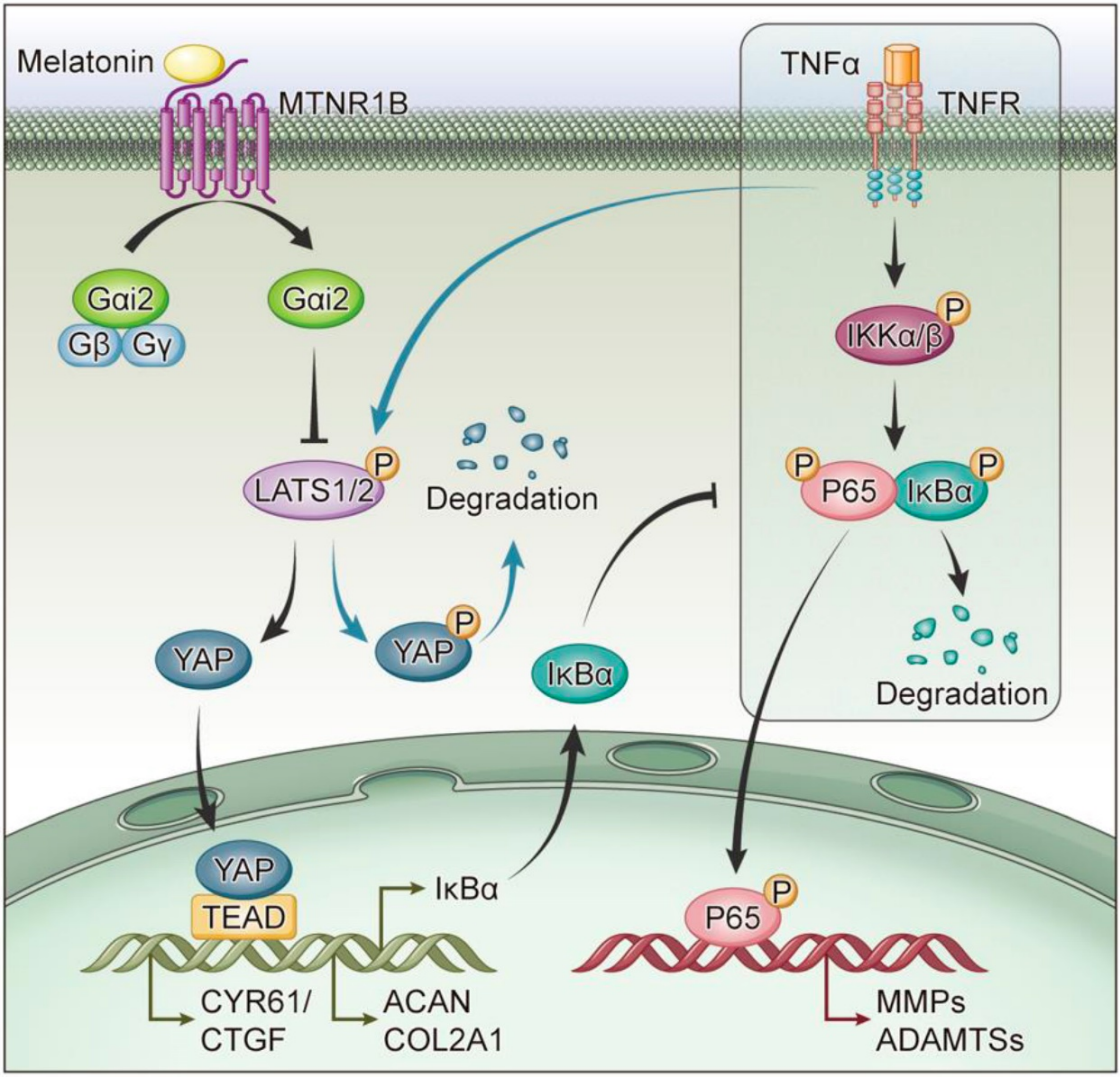
** Schematic of melatonin's protective effect on NP cells in the presence of TNF-α.** Melatonin upregulates YAP protein levels via activating MTNR1B, which subsequently recruits Gαi2 protein. YAP upregulated by Gαi2 activation results in anabolic enhancement of NP cells (left). Meanwhile, melatonin-mediated YAP upregulation increases the expression of IκBα and suppresses the TNF-α-induced activation of NF-κB pathway (right), which thereby inhibiting the catabolism of NP cells.

G proteins are the major downstream components of GPCRs. MTNR1B always coupled to Gαi/o protein, including Gαi1, Gαi2 and Gαi3, to regulate multiple downstream signaling pathways^38^. When MTNR1B is activated in NP cells, which G protein it couples to is still unknown. In our study, the endogenous immunoprecipitation assay showed that when MTNR1B was inactivated, there were few G protein coupled to MTNR1B (Figure 7A). However, once MTNR1B was activated by the treatment of melatonin, it could significantly bind to Gαi2 (Figure 7B), suggesting Gαi2 plays a predominant role in MTNR1B signaling in NP cells. Subsequently, we examined whether Gαi2 mediated the melatonin-induced protective effects in NP cells. We found that Gαi2 silencing (Figure 7C and D) could significantly reverse the effects of melatonin on restoring the metabolism of NP cells (Figure 7E and F). Furthermore, as shown in Figure 6G, Gαi2 silencing could promote the phosphorylation of YAP and p65 in NP cells, which antagonized the downregulation of melatonin. In addition, Gαi2 silencing could decrease nuclear translocation of YAP, as well as promote the nucleus translocation of p65 (Figure 7F and G). We speculated that the loss of Gαi2 cuts off the link between melatonin and YAP, which leads to the restoration of NF-κB pathway activity. Based on our results, it suggests that melatonin can bind to MTNR1B and upregulate the protein expression of YAP by recruiting Gαi2. YAP upregulation not only promotes the anabolism of NP cells, but also increases the expression of IκBα and inhibits the NF-κB signaling pathway, thereby inhibiting the catabolism of NP cells (Figure 8). In this way, melatonin can achieve the purpose of regulating the matrix metabolism of human NP cells and delaying IDD.

## Discussion

In this study, we present several lines of evidence supporting the protective role of melatonin and YAP in restoring TNF-α-induced metabolic disturbance of human NP cells: (1) melatonin can reverse TNF-α-impaired metabolism of NP cells via activating MTNR1B, which subsequently recruits Gαi2; (2) YAP upregulation by activation of Gαi2 results in increased anabolism and decreased catabolism of NP cells by increasing the expression of IκBα thereby suppressing the NF-κB pathway. Our results suggest that melatonin may be considered as a potential therapeutic drug for the treatment of IDD.

TNF-α, which involved in multiple pathological changes of IDD, is considered to be the key mediator of IDD and LBP^39^. Of note, TNF-α not only induces the expression of MMPs and ADAMTSs to destroy the ECM, but also amplifies the inflammatory responses in NP cells by inducing the secretion of several inflammatory mediators, including CCL2, CCL3, NO and PGE2^10^, ^40^, ^41^. The senescence, apoptosis and pyroptosis of NP cells induced by TNF-α are also important reasons for the development of IDD^39^, ^42^. These pathological changes eventually lead to the deterioration of IDD. In the present study, we found that TNF-α was upregulated in the degenerative NP tissues (Figure 3). And we found that TNF-α can promote the expression of MMPs and ADAMTSs by activating the NF-κB pathway. On the other hand, TNF-α treatment also can activate the Hippo pathway and inhibit the activity of YAP, thereby affecting the anabolism of NP cells. Besides, we found that TNF-α treatment unregulated the expression of inflammatory mediators, such as CCL2, TNF-α and IL-1β. Our results demonstrated the important role of TNF-α in the IDD progression.

Melatonin has been proven to have an important protective effect on a variety of orthopedic degenerative diseases, including osteoporosis and osteoarthritis 21, 43. Melatonin also has a protective effect on NP cells, which alleviate the development of IDD. Although relevant studies have confirmed that melatonin can protect NP cells under the action of IL-1β^44^, ^45^, few studies have addressed the protective effect of melatonin on NP cells in the presence of TNF-α. In our study, we found that melatonin can reverse TNF-α-induced metabolic disturbance of NP cells (Figure 3). Melatonin was found to activate the membrane receptor, MTNR1B (Figure 4), subsequently recruiting the Gαi2 protein in NP cells (Figure 7). Furthermore, we found that YAP activity was maintained by melatonin treatment in the presence of TNF-α (Figure 5). Melatonin-induced YAP upregulation ultimately results in suppression of NF-κB signaling and restoration of the metabolism imbalance of NP cells (Figures 5 and 6). In other words, melatonin can protect matrix metabolism of NP cells against TNF-α by the mechanism involving membrane receptors, G protein, Hippo/YAP signaling pathway and NF-κB signaling pathway (Figure 8).

In recent studies, the Hippo/YAP pathway, which is closely related to mechanical stress, has been shown to be important in bone and cartilage diseases^46^. Zhou et al.^47^ reported that YAP dysfunction leads to bone fracture in the Piezo2 CKO mice. Deng et al.^28^ showed that loss of YAP exaggerates cartilage degradation during osteoarthritis. A study by Fu et al.^48^ revealed that YAP can reverse senescence of chondrocytes and alleviate the progression of osteoarthritis by upregulating the expression of FOXD1. However, the role of YAP in IDD is still controversial. Zhang et al.^49^ showed that YAP expression levels decrease with age in intervertebral disc. Another study by Zhang et al.^50^ revealed that YAP inhibition significantly induced senescence in NP cells. By contrast, Chen et al.^51^ showed that YAP could be activated by IL-6 and involved in degeneration of NP cells. Therefore, the exact role of YAP in IDD needs further research. In the present study, we found that the nuclear translocation of YAP was maintained by melatonin in the presence of TNF-α, which eventually restored the metabolism imbalance of NP cells and alleviated IDD. Our study verified the protective role of YAP against the inflammation in NP cells. This will provide a new therapeutic target for treatment of IDD.

In most instances, melatonin activates two high-affinity G protein-coupled receptors, MTNR1A and MTNR1B, to exert its biological functions in mammal^38^. Melatonin receptor signaling pathways have been found to be related to several diseases, such as type II diabetes mellitus^52^, multiple sclerosis^53^ and tumor recurrence^54^. Our previous studies also showed that melatonin receptor signaling pathways play an important role in osteogenesis and chondrogenesis of bone marrow mesenchymal stem cells^16^, ^18^, suggesting the importance of melatonin receptor pathways in the development and repair of bone and cartilage. In the current study, we explored the expression of MTNR1A and MTNR1B in human normal and degenerative NP tissues, and showed that the expression of MTNR1A and MTNR1B was both downregulated in degenerative NP tissues, which is consistent with a previous study by Li et al.^25^ Furthermore, we verified that the activation of MTNR1B, but not MTNR1A, mediated the protective effects of melatonin in NP cells (Figure 4). In general, MTNR1A and MTNR1B have similar physiological roles. However, previous studies have shown that the MTNR1B seems to be more related to the musculoskeletal system^55^, ^56^. Combined with our study, we speculate that MTNR1B may be a melatonin receptor closely related to the musculoskeletal system. In addition, we found that MTNR1B specifically binds to Gαi2 protein, thereby maintaining the protein activity of YAP, which mediated the protective effects of melatonin in NP cells. Interestingly, our previous study showed that MTNR1B can also interact with Gαi2 in the chordoma cell lines, U-CH1 and MUG-CC1^54^. We speculate that this coincidence may be related to the homology between NP cells and chordoma cell lines^57^.

LBP, which has always been associated with IDD, is still a worldwide health problem. In the early stage of the disease, LBP is usually treated with nonsteroidal anti-inflammatory drugs (NSAIDs) and physical therapy. If unsuccessful, invasive surgical procedures are always under consideration as a late resort. However, the existing treatments do not counteract the process of IDD. Currently, restoring the homeostasis of anabolic and catabolic activities in NP tissues is still a worldwide challenge. Multiple approaches have been used for the regeneration of intervertebral discs, including anti-catabolic and anti-inflammatory strategies, pro-anabolic molecules, cell supplementation and biomaterials^58^. However, the safety and effectiveness of these approaches still need to be further clarified. Melatonin, a hormone secreted by the pineal gland, has good safety and little side effects. Consistent with previous studies, our results showed that melatonin also has a protective effect on the metabolism of NP cells, thereby attenuating IDD progression. Unlike other cytokine therapies that only have a single therapeutic effect, melatonin not only has anti-inflammatory effects, but also has pro-anabolic effects, ensuring that it can protect the NP cells at multiple levels. Moreover, the dysregulation of rhythm-related gene expression can lead to acceleration of IDD^59^, which demonstrates that melatonin, a hormone that regulates the circadian rhythm, may also play a protective role in IDD. In addition, the pro-sleep and anti-depression effects of melatonin can also help relieve the symptoms of IDD patients. Taking all considerations together, we demonstrated that melatonin may be a potential therapeutic drug for the treatment of IDD.

## Conclusion

In conclusion, our study showed the role of melatonin on restoring matrix metabolism of NP cells in the presence of TNF-α. The present study also revealed that MTNR1B/Gαi2/YAP axis plays an essential part in the effects of melatonin on NP cells. Therefore, our results provided more theoretical basis for melatonin as a candidate drug for the treatment of IDD.

## Supplementary Material

Supplementary figures and table.

## Abbreviations

LBP: Low back pain
IDD: Intervertebral disc degeneration
NP: Nucleus pulposus
TNF-α: Tumor necrosis factor-α
IL-1β: Interleukin-1β
MTNR1A: Melatonin receptor 1A
MTNR1B: Melatonin receptor 1B
ECM: Extracellular matrix
COL2A1: Collagen type II
ACAN: Aggrecan
MMP13: Matrix metalloproteinase 13
ADAMTS5: A disintegrin and metalloproteinase with thrombospondin motifs 5
YAP: Yes-associated protein
LAST1/2: Large tumor suppressor 1/2
CYR61: Cysteine rich angiogenic inducer 61
CTGF: Connective tissue growth factor
NF-κB: Nuclear factor‐κB
IκBα: NF-κB inhibitor alpha
MMP9: matrix metalloproteinase 9
CCL2: C-C motif chemokine ligand 2
Gαi2: G protein subunit alpha i2
CHX: Cycloheximide
NC: Negative control
shRNA: short hairpin RNA
Ub: Ubiquitin
RT-qPCR: Reverse transcription‑quantitative polymerase chain reaction
IHC: Immunohistochemistry
IF: Immunofluorescence
