# Supplementary material for: Melatonin reverses tumor necrosis factor-alpha-induced metabolic disturbance of human nucleus pulposus cells via MTNR1B/Gαi2/YAP signaling
Source: Int J Biol Sci. 2022 Mar 6;18(5):2202–19. doi: 10.7150/ijbs.65973 (PMC8935230; doi:10.7150/ijbs.65973)
Supplement: Supplementary file 1 — Supplementary figures and table. [file ijbsv18p2202s1.pdf]

Table I. Clinical information of patients

Case	Sex	Age	Affected IVD level	Pfrrimann
1	Male	56	L4/5	4
2	Female	56	L4/5	5
3	Female	56	L4/5	3
4	Male	54	L5/S1	5
5	Female	55	L5/S1	4
6	Male	54	L4/5	4
7	Male	50	L5/S1	5
8	Female	39	L5/S1	5
9	Female	63	L4/5	5
10	Male	60	L4/5	5
11	Male	47	L4/5	3
12	Female	53	L4/5	4
13	Male	14	L4/5	1
14	Female	15	L3/4	1
15	Male	25	L3/4	2
16	Female	16	L5/S1	2
17	Male	21	L3/4	2
18	Female	18	T12/L1	2

Table II. All primers for reverse transcription-quantitative polymerase chain reaction

Gene	Primer sequence(5'-3')
GAPDH	Forward: AGAAAAACCTGCCAAATATGATGAC;
	Reverse: TGGGTGTCGCTGTTGAAGTC;
MTNR1A	Forward: ATGTTTCCTGCGTTCCTGAG;
	Reverse: CTAGCCTGCGTCCTCATCTT;
MTNR1B	Forward: GGAACGCAGGTAATTTGTTCTTG;
	Reverse: TTAGCGGGTAGGGGTAGAAGG;
COL2A1	Forward: GGCAATAGCAGGTTCACGTACA
	Reverse: CGATAACAGTCTTGCCCCACTT
ACAN	Forward: TGCATTCCACGAAGCTAACCTT;
	Reverse: GACGCCTCGCCTTCTTGAA;
MMP13	Forward: ACTGAGAGGCTCCGAGAAATG;
	Reverse: GAACCCCGCATCTTGGCTT;
ADAMTS5	Forward: GAACATCGACCAACTCTACTCCG;
	Reverse: CAATGCCCACCGAACCATCT;
YAP	Forward: TAGCCCTGCGTAGCCAGTTA;
	Reverse: TCATGCTTAGTCCACTGTCTGT;
CYR61	Forward: CTCGCCTTAGTCGTCACCC;
	Reverse: CGCCGAAGTTGCATTCCAG;
CTGF	Forward: CAGCATGGACGTTCGTCTG;
	Reverse: AACCACGGTTTGGTCCTTGG;
MMP9	Forward: TGTACCGCTATGGTTACACTCG;
	Reverse: GGCAGGGACAGTTGCTTCT;
CCL2	Forward: CAGCCAGATGCAATCAATGCC;
	Reverse: TGGAATCCTGAACCCACTTCT;
GNAI2	Forward: TACCGGGCGGTTGTCTACA;
	Reverse: GGGTCGGCAAAGTCGATCTG;
TNF-α	Forward: CCTCTCTCTAATCAGCCCTCTG;
	Reverse: GAGGACCTGGGAGTAGATGAG;
IL-1β	Forward: ATGATGGCTTATTACAGTGGCAA;
	Reverse: GTCGGAGATTCGTAGCTGGA;

GAPDH, glyceraldehyde-3-phosphate dehydrogenase; MTNR1A, melatonin receptor 1A; MTNR1B, melatonin receptor 1B; COL2A1, collagen type II ; ACAN, aggrecan; MMP13, matrix metalloproteinase 13; ADAMTS5, a disintegrin and metalloproteinase with thrombospondin motifs 5; YAP, yes-associated protein; CYR61, cysteine rich angiogenic inducer 61; CTGF, connective tissue growth factor; IκBα, NFκB inhibitor alpha; MMP9, matrix metalloproteinase 9; CCL2, C-C motif chemokine ligand 2; GNAI2, G protein subunit alpha i2; TNF-α, tumor

necrosis factor-α; IL-1β, interleukin-1β.

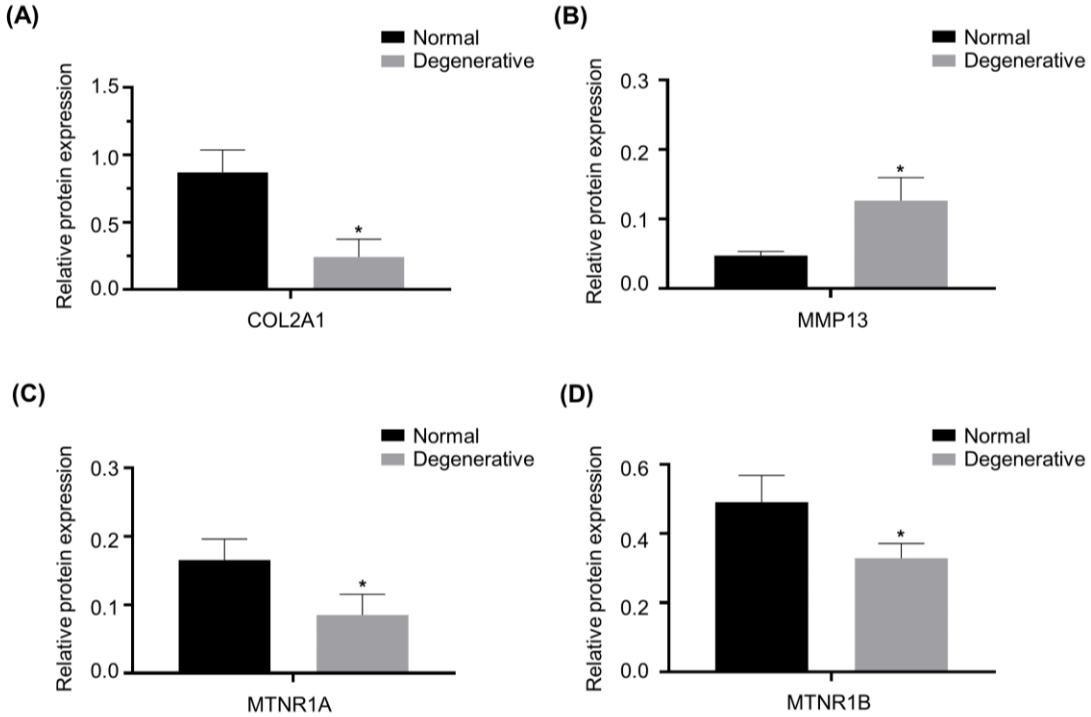

Supplementary Figure 1. Quantification of protein expression in human NP tissues. WB Quantification of **(A)** COL2A1, **(B)** MMP13, **(C)** MTNR1A, and **(D)** MTNR1B in normal and degenerative human NP tissues. *$P < 0.05$.

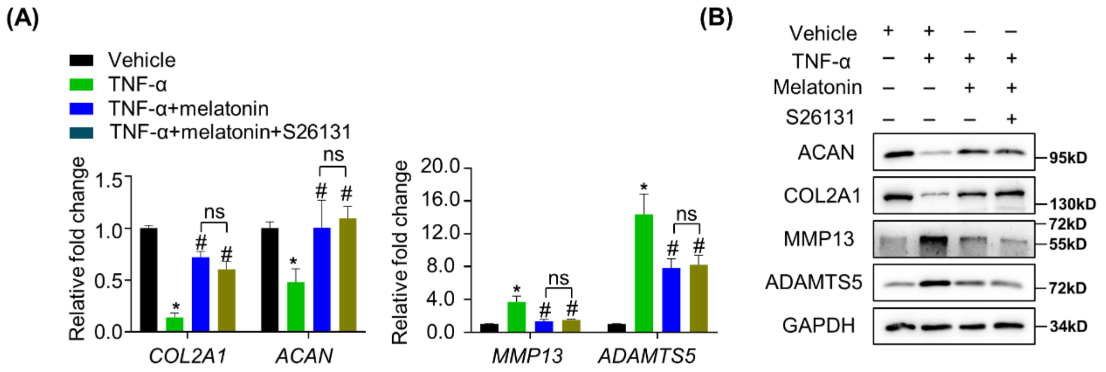

Supplementary Figure 2. MTNR1A antagonist cannot block melatonin's protective effects on NP cells. RT-qPCR **(A)** and WB **(B)** analyses were performed to assess the expression of COL2A1, ACAN, MMP13, and ADAMTS5 in human NP cells with or without pretreatment of MTNR1A antagonist S26131 (5 μM) for 1 hour followed by the indicated treatment for 48 hours. * means $P < 0.05$ compared with the vehicle group. # means P < 0.05 compared with the TNF-α group. Ns means not significant.

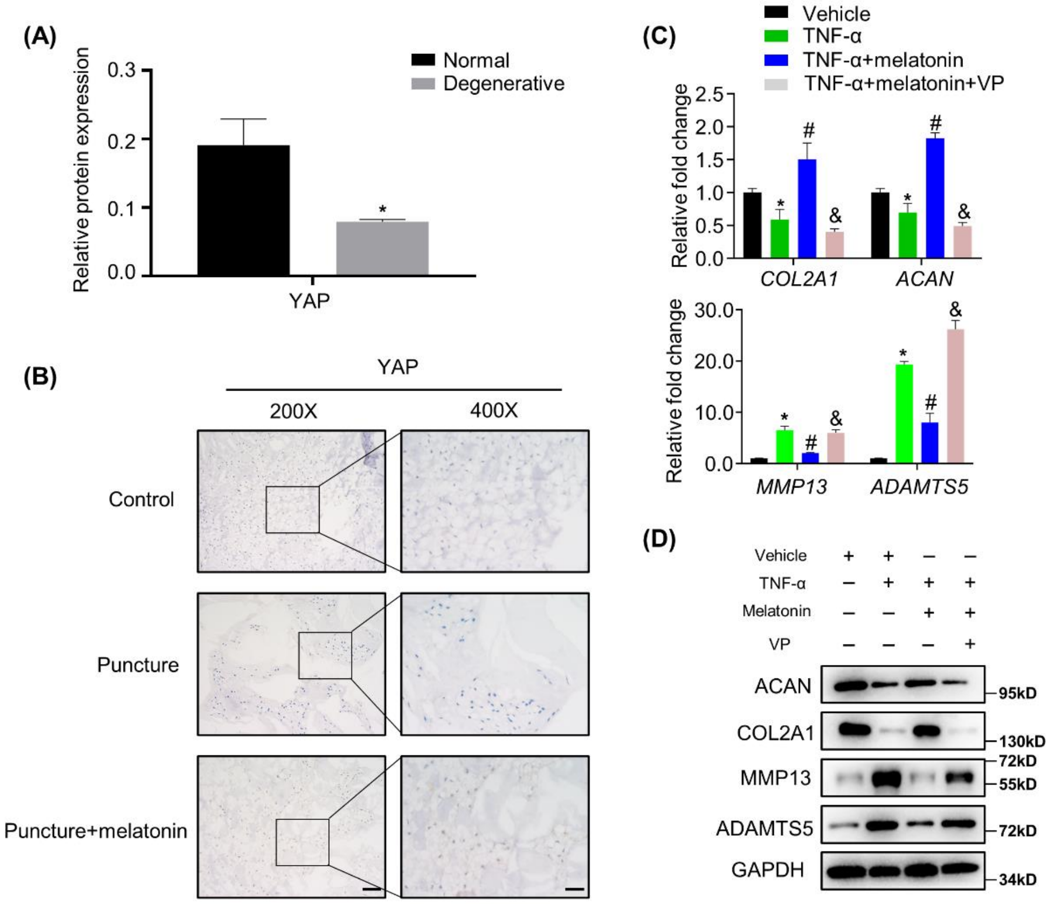

Supplementary Figure 3. YAP mediates melatonin-induced protective effects on NP cells in the presence of TNF-α. (A) WB Quantification of YAP in normal and degenerative human NP tissues. **(B)** The expression of YAP in rat NP tissues followed by treatment with or without puncture and melatonin were assessed by IHC. Representative images of different magnifications were displayed. Scale bars: 100 μm and 50 μm for 200× and 400× images, respectively. RT-qPCR **(B)** and WB **(C)** analyses were applied to assess the expression of COL2A1, ACAN, MMP13, and ADAMTS5 in human NP cells with or without the treatment of vehicle (ethanol), TNF-α (10 ng/ml), melatonin (100 μM) and VP (2.5 μM) for 48 hours. * means $P < 0.05$ compared with the vehicle group. # means $P < 0.05$ compared with the TNF-α group. & means $P <$ 0.05 compared with the TNF-α and melatonin group.

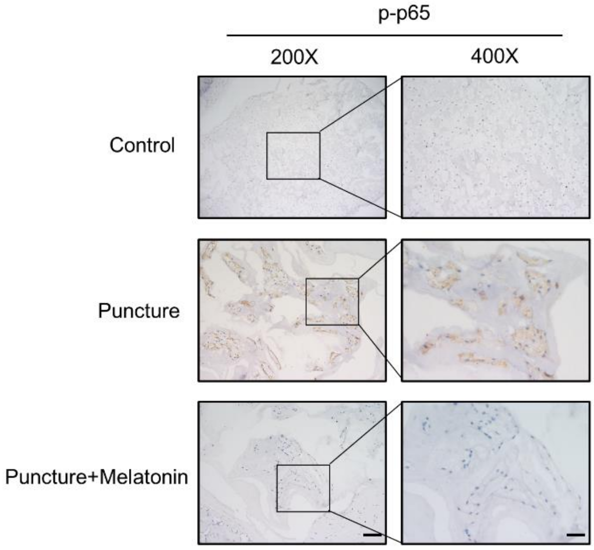

Supplementary Figure 4. Melatonin-mediated YAP upregulation attenuates TNF-α-induced NF-κB pathway activation by enhancing the expression of IκBα protein. The expression of p-p65 in rat NP tissues followed by treatment with or without puncture and melatonin was assessed by IHC. Representative images of different magnifications were displayed. Scale bars: 100 μm and 50 μm for 200× and 400× images, respectively.
